# Multimodal imaging of indapamide-induced bilateral choroidal effusion: a case report

**DOI:** 10.1186/s12886-021-02147-3

**Published:** 2021-11-04

**Authors:** Shizuka Takahashi, Shinichi Usui, Noriyasu Hashida, Hiroshi Kubota, Kentaro Nishida, Hirokazu Sakaguchi, Kohji Nishida

**Affiliations:** 1 Department of Ophthalmology, Osaka University Graduate School of Medicine, 2-2 Yamadaoka, Suita, Osaka 565-0871 Japan; 2 Department of Advanced Device Medicine, Osaka University Graduate School of Medicine, 2-2 Yamadaoka, Suita, Osaka 565-0871 Japan; 3 Department of Ophthalmology, Gifu University Graduate School of Medicine, Yanagido, Gifu, Japan; 4 Integrated Frontier Research for Medical Science Division, Institute for Open and Transdisciplinary Research Initiatives (OTRI), Osaka University, 2-2 Yamadaoka, Suita, Osaka 565-0871 Japan

**Keywords:** Acute angle-closure glaucoma, Ciliary body edema, Drug-induced choroidal effusion, Indapamide, Multimodal imaging, Myopia

## Abstract

**Background:**

Indapamide, a sulfonamide diuretic used to treat hypertension, has been reported to have ocular side effects of acute angle-closure glaucoma, transient myopia and choroidal effusion whose immediate etiology is uncertain. This report aims to clarify the nature of indapamide-induced edema of the entire eyeball using multimodal imaging.

**Case presentation:**

A 60-year-old woman who was following a long-term carbohydrate-restricted diet and receiving oral treatment for hypertension was referred to our department for eye pain. Indapamide (1 mg daily) was prescribed for uncontrolled hypertension 5 days before her visit; she took the medication for only 3 days and then stopped due to dry eye. However, she began to feel eye pain the day after her last dose, and the pain gradually intensified. She experienced no decrease in visual acuity at the initial visit; however, an extremely shallow anterior chamber was observed in both eyes, along with a slight increase in intraocular pressure. For differential diagnosis, ocular manifestations were evaluated with wide-field fundus photography, optical coherence tomography (OCT) of both anterior and posterior segments, fluorescein / indocyanine green angiography, ultrasound biomicroscopy and head magnetic resonance, showing edema of the entire eyeball. Treatment with tropicamide and phenylephrine hydrochloride drops resulted in rapid recovery of the anterior chamber depth and disappearance of the choroidal effusion within 3 days.

**Conclusions:**

Multimodal imaging is useful for diagnosing drug-induced choroidal effusion by evaluating ocular conditions before and after treatment.

## Background

Various drugs have been reported to induce angle-closure glaucoma and transient myopia [1]. Indapamide is one such drug. There have been several reports of transient myopia, acute angle-closure glaucoma, and choroidal effusion occurring as adverse effects of indapamide, but the underlying mechanisms remain unclear; three candidate mechanisms have been proposed in the literature [2–5]: (1) osmotic disturbances of the lens that increase its thickness and change its refractive index, (2) ciliary body edema, and (3) accommodative spasm of the ciliary muscles. The present case report aimed to reveal the characteristics of indapamide-induced choroidal effusion through multimodal imaging; the evidence supported the second of the three mechanisms presented above.

## Case presentation

A 60-year-old woman was referred to our outpatient clinic because of eye pain. She was on a long-term carbohydrate-restricted diet and had been taking hypertension medication for several years. She had no history of ophthalmic disease other than high myopia. Her most recent systolic and diastolic blood pressure values were 140–180 mmHg and 100–120 mmHg, respectively; thus, 1 mg/day of a thiazide diuretic (indapamide) was prescribed along with pravastatin sodium and amlodipine besylate. She took indapamide (1 mg) for 3 days but then stopped taking it because her eyes had begun to feel dry. The next night, she experienced dimmed vision, eye pain, and photophobia upon standing up from her bed; the following day, she visited a local ophthalmologist. Her intraocular pressure (IOP) was 26 mmHg in the right eye (OD) and 29 mmHg in the left eye (OS). She was diagnosed with angle-closure glaucoma and was prescribed topical medications, which she did not apply. As the symptoms did not improve, she subsequently visited another ophthalmologist. The IOP was 24 mmHg in OD and 25 mmHg in OS. Vogt-Koyanagi-Harada disease was suspected because of the shallow anterior chamber depth (ACD). She was referred to our department on the same day.

**Fig. 1:**
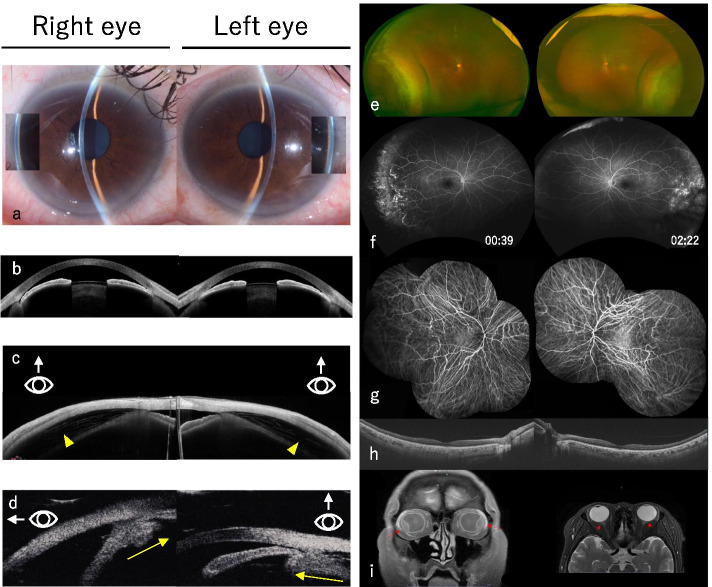
Multimodal images in the acute phase. **a** Slit-lamp microscopy revealed narrowing angles. **b** Cornea/anterior segment optical coherence tomography. The anterior chamber depths of both eyes were extremely shallow. Relative miosis by the suspected effect of prostaglandin E2 was observed. **c** Optical coherence tomography of the superior side of the cornea/anterior segment showed choroidal effusion between the retina and sclera (yellow arrowheads). This held true in all quadrants. **d** Ultrasound biomicroscopy revealed forward displacement of the lens and the edematous ciliary body (yellow arrows). **e** Color fundus photograph demonstrating symmetrical and bilateral choroidal effusion. **f** Fluorescein angiography showed vessel leakage, granular hyperfluorescence in the peripheral retina, and a small area of nonperfusion corresponding to the area of choroidal effusion. **g** Indocyanine green revealed no significant findings in the temporal lesions compared to fluorescein angiography. **h** Optical coherence tomography showed a thickened choroid with choroidal vascular dilation around the posterior pole. **i** T1 weighted image of magnetic resonance imaging scan of the head (left) showing a high-signal area on the surface and in the deep layers of liquid (red arrows). T2 weighted image (right) showing a small amount of fluid accumulated in the sub-Tenon’s space; a small amount of edema was suspected around the optic nerve head (red arrowheads)

Her best-corrected visual acuity was 20/20 with − 9.00 D sph, − 0.75 D cyl × 20 correction in OD and 20/16 with − 8.75 D sph, − 1.25 D cyl × 150 correction in OS. The IOP measured 24 mmHg and 32 mmHg in OD and OS, respectively (Table 1). Pupillary reactions were normal. Slit-lamp examination revealed no inflammation in the shallow anterior chamber of either eye. A laser flare meter showed no inflammation. The axial length and ACD measured centrally by optical coherence tomography (OCT) of the cornea/anterior segment were 26.47 mm and 1.340 mm, respectively, in OD and 26.42 mm and 1.276 mm in OS (Fig. 1a, b, Table 1). The central corneal thickness (CCT) and lens thickness (LT) were slightly thickened without any difference between OD and OS compared to the prominently swollen ciliary body and choroid. Specifically, the CCT was 527 μm for OD and 520 μm for OS, and the LT was 4.407 mm for OD and 4.427 mm for OS (Table 1). The OCT of the cornea/anterior segment revealed choroidal effusion (Fig. 1c) due to the forward displacement of the lens and the edematous ciliary body (Fig. 1d). A fundus examination revealed extensive, symmetrical, bilateral choroidal effusion on the temporal side (Fig. 1e). Fluorescein angiography (FA) showed granular hyperfluorescence, tortuous vessels, vascular leakage, a nonperfusion area corresponding to the site of choroidal effusion on the temporal side, and retinal folds toward the posterior pole in both eyes (Fig. 1f), whereas indocyanine green revealed no significant findings (Fig. 1g). The macula was intact on OCT, but the choroid was significantly thickened to 342 μm in OD and 316 μm in OS (Fig. 1h, Table 1). Head magnetic resonance imaging (MRI) revealed no intracranial lesions but indicated edema of the entire eyeball. In other words, T1 weighted image showed a high-signal area on the surface and in the deep layers of liquid. Fluid had accumulated symmetrically on the sclera, with a high signal on both temporal sides. T2 weighted image showed a small amount of fluid accumulated in the sub-Tenon’s space; a small amount of edema was suspected around the optic nerve head (Fig. 1i). Blood tests revealed no inflammation or electrolyte abnormality and no evidence of any infectious diseases or autoantibodies.

**Table 1:** Biometric data of the anterior segments of the right/left eyes acquired from optical coherence tomography of both anterior and posterior segments

	Presentation Day	Day 1 (1st Day of Eye Drops	Day 2	Day 3	1 Week	2.5 Months
VA	20/20, 20/16	–	–	–	20/20, 20/20	20/16, 20/16
Sphere (Diopters)	−9.00 / −8.75	–	–	–	−7.50 / −6.75	− 7.50 / −7.00
AL (mm)	26.47 / 26.42	–	–	–	–	26.60 / 26.54
IOP (mmHg)	24 / 32	20 / 27	14 / 15	13 / 16	18 / 19	18 / 18
CCT (μm)	527 / 520	523 / 518	510 / 506	515 / 491	506 / 503	514 / 508
ACD (mm)	1.340 / 1.276	1.549 / 1.502	1.953 / 2.008	2.195 / 2.312	2.649 / 2.616	2.559 / 2.497
LT (mm)	4.407 / 4.427	4.343 / 4.453	4.379 / 4.436	4.383 / 4.435	4.259 / 4.287	4.255 / 4.296
CT (μm)	342 / 316	–	–	294 / 221	189 / 136	142 / 136

*Day 1* First day of treatment, *VA* Visual acuity, *AL* Axial length, *IOP* Intraocular pressure, *CCT* Central corneal thickness, *ACD* Anterior chamber depth, *LT* Lens thickness, *CT* Choroidal thickness

**Fig. 2:**
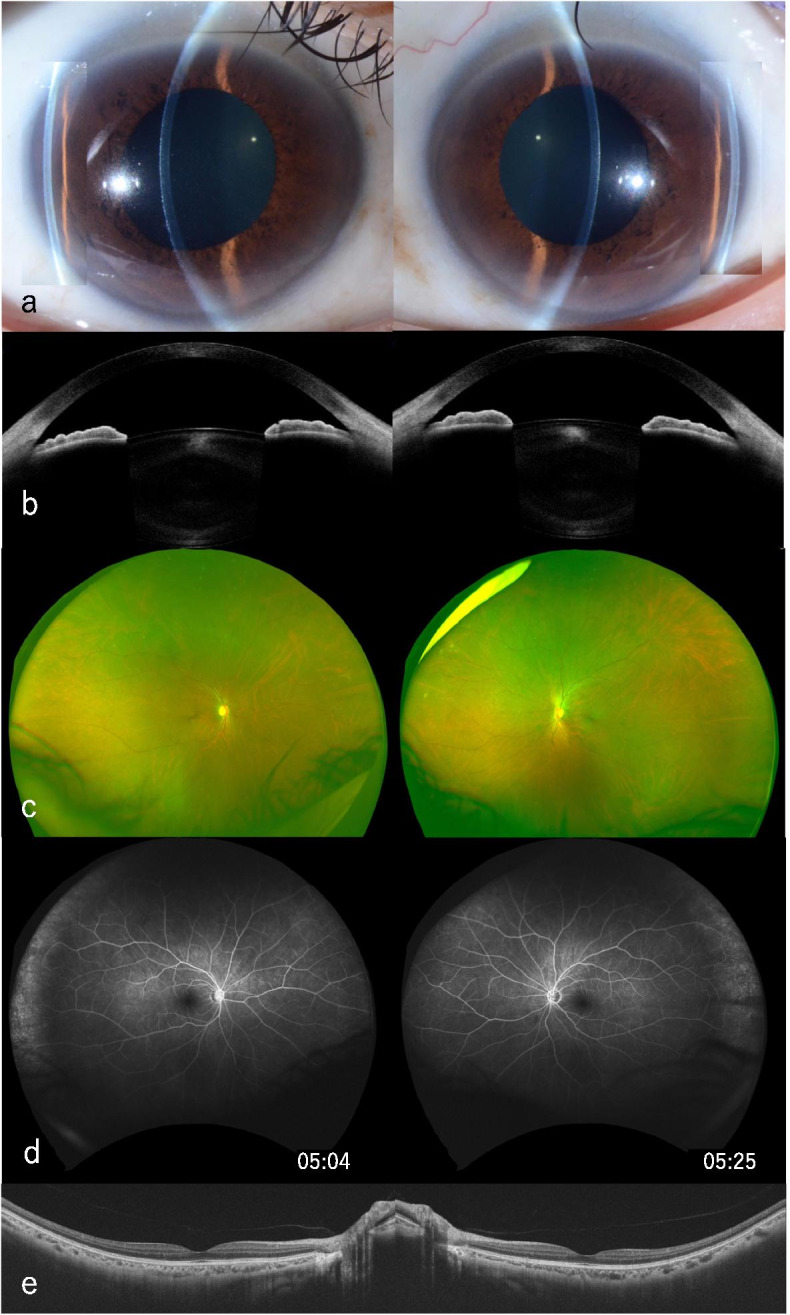
Multimodal images in the convalescence phase. **a** Slit-lamp microscopy revealed that the anterior chamber depth was restored. **b** Cornea/anterior segment optical coherence tomography. In contrast to the acute phase, the anterior chamber reached a sufficient depth, and the pupils showed no evidence of miosis by the effect of prostaglandin E2. **c** Color fundus photography showed that the choroidal effusions had disappeared. **d** Fluorescein angiography after 2.5 months. Leakage from blood vessels disappeared, peripheral granular hyperfluorescence improved, no area of nonperfusion was observed, and the choroidal thickness returned to normal. **e** Optical coherence tomography showed that the choroid returned to its normal thickness

Based on the above findings, we considered the possibility that edema of the ciliary body may have mainly contributed to the forward displacement of the lens. Administration of 0.5% tropicamide and 0.5% phenylephrine hydrochloride (Mydrin-P; Santen, Osaka, Japan) drops and 1% betamethasone three times per day was initiated; by the second day of treatment, the IOP had decreased to 14 mmHg and 15 mmHg in OD and OS, respectively. After 3 days of treatment, the ACD gradually deepened to more than 2.0 mm, and the IOP normalized (Fig. 2a-b, Table 1). One week after the start of treatment, the CCT improved to 506 μm for OD and 503 μm for OS, and the LT improved to 4.259 mm for OD and 4.287 mm for OS (Table 1). Meanwhile, the bilateral choroidal effusion on the inferotemporal side disappeared (Fig. 2c). The eye pain resolved, as did the other optical symptoms. On the seventh day of treatment, myopia was reduced to 20/20 with − 7.50 D sph in OD and 20/20 with − 6.75 D sph, − 0.75 D cyl × 120 correction in OS, and the patient was discharged. After 2.5 months, follow-up FA revealed that the vascular leakage and peripheral granular hyperfluorescence had improved, and no area of nonperfusion was observed (Fig. 2d). These findings suggest that vasodilation and circulatory deficiency were reversibly improved. The choroid returned to its normal thickness of 142 μm for OD and 136 μm for OS, as did other segments (Fig. 2e, Table 1).

## Discussion and conclusions

We were able to evaluate the entire eyeball using the latest imaging equipment in a case of choroidal effusion induced by indapamide. The methods we used allowed an accurate diagnosis. In cases diagnosed as acute angle-closure glaucoma, characterized by elevated intraocular pressure and a narrow angle, the differential diagnosis is critical because cataract surgery is an invasive treatment. Uveal effusion syndrome is included in the differential diagnosis of choroidal effusion and is easily ruled out in this case because microphthalmia and scleral hypertrophy are detected by MRI and B mode ultrasound. Myopia, another condition that was present in this patient, is observed in most cases of drug-induced choroidal effusion [6]. Multimodal imaging revealed that the entire eyeball was edematous in this case and helped us arrive at an accurate diagnosis.

However, the reasons for the extension of edema in the eye are still unclear. According to previous reports, it has been speculated that people who are sensitive to drugs may respond to them with vasodilation and increased vascular permeability due to increased synthesis of prostaglandin E2 [2, 4]. Prostaglandin E2 increases the protein content of the aqueous humor, leading to an increase in IOP [7]. Since the cornea and lens are avascular tissues, they did not edema much even when exposed to prostaglandin E2, and the ciliary body and choroid are richly vascularized tissues and can easily become edematous. Thus, our case supports the mechanism by which ciliary edema causes the lens to move anteriorly, resulting in angle-closure glaucoma. Circulatory failure was suggested by the FA findings of vasodilation, leakage, and an area of nonperfusion corresponding to the choroidal effusion lesion, as well as the OCT finding of choroidal thickening [3]. Choroidal effusion was caused by blood stasis.

The question that arises based on previous reports and this case is why drug-induced choroidal effusion tends to appear in the temporal region [3]. We can speculate about a possible answer based on anatomical knowledge. Prostaglandin E2 production increases in response to indapamide [2, 4], and the venous drainage of arteries, especially long posterior ciliary arteries, is markedly congested [8], causing vasodilation and tissue edema. In the sclera and choroid, stagnant blood may cause choroidal effusion; myopia further increases their susceptibility to this effect because of the thinner sclera and choroid. Furthermore, in normal eyes, the choroidal vessels on the temporal side are coarser than those on the nasal side [9], and an inadequate temporal venous drain during circulatory failure results in choroidal effusion on the temporal side of the retina rather than on the nasal side. This is the difference between drug-induced choroidal effusion and circumferential choroidal effusion with hypotony because the eyeball cannot maintain its shape.

Multimodal imaging in this case gave us new information on pathology and was useful to confirm that the severity of edema differed depending on the parts of the eyeball and to reach the accurate diagnosis. MRI revealed that the entire eyeball was edematous in the case of indapamide-induced bilateral choroidal effusion. Based on the fact, a detailed history taken along with basic imaging investigations, such as OCT, B-mode and UBM, might be enough to diminish differential diagnosis and the management because all to do in daily practice is to confirm that the severity of edema differs depending on the parts of the eyeball. However, wider-view fundus photography and OCTs of both anterior and posterior segments are effective in detecting lesions in situations where mydriasis is difficult such as this case, acute angle-closure glaucoma or Vogt-Koyanagi-Harada disease which should be ruled out. On the other hand, UBM examination is quite invasive. Our patient actually refused it after treatment. It is difficult to use UBM for a follow-up examination. The OCT of the cornea/anterior segment gives us a lot of information non-invasively at once which is the condition of the ciliary body and anterior choroid, the corneal thickness, lens thickness, and anterior chamber depth. B-mode is also effective to detect choroidal detachment, but it is low resolution. The states of both retina and choroid can be investigated with high resolution posterior OCT. Thus, MRI is not mandatory every time, but OCTs of both anterior and posterior segments and wide-angle fundus photograph are able to show edema of the entire eyeball which indicates the pathology of indapamide-induced bilateral choroidal effusion. They enable to be an accurate diagnosis and a consecutive management.

In conclusion, we presented a case of acute angle-closure and choroidal effusion induced by indapamide intake; these conditions were accurately diagnosed using the latest equipment. The mechanisms are still unclear and need to be elucidated in future studies. Using multimodal imaging, however, we showed edema of the entire eyeball, which was useful for differential diagnosis, and we evaluated the ocular conditions before and after treatment.

## Data Availability

All data generated or analyzed during this study are included in this published article.

## Abbreviations

ACD: Anterior chamber depth
CCT: Central corneal thickness
FA: Fluorescein angiography
IOL: Intraocular lens
IOP: Intraocular pressure
LT: Lens thickness
MRI: Magnetic resonance imaging
OCT: Optical coherence tomography
OD: Right eye
OS: Left eye
